# Determination of the 3D Human Spine Posture from Wearable Inertial Sensors and a Multibody Model of the Spine

**DOI:** 10.3390/s22134796

**Published:** 2022-06-24

**Authors:** Florian Michaud, Urbano Lugrís, Javier Cuadrado

**Affiliations:** Laboratory of Mechanical Engineering, University of La Coruña, 15403 Ferrol, Spain; urbano.lugris@udc.es (U.L.); javier.cuadrado@udc.es (J.C.)

**Keywords:** spinal disorders, injury prevention, motion capture, inertial sensor

## Abstract

Determination of spine posture is of great interest for the effective prevention, evaluation, treatment and evolution monitoring of spinal disorders. Limitations of traditional imaging systems, including cost, radiation exposure (for X-ray based systems), projection volume issues and subject positioning requirements, etc., make non-invasive motion assessment tools effective alternatives for clinical and non-clinical use. In this work, a procedure was developed to obtain a subject-specific multibody model of the spine using either inertial or optical sensors and, based on this multibody model, to estimate the locations and orientations of the 17 vertebrae constituting the thoracolumbar spine. The number and calibration of the sensors, angular offsets, scaling difficulties and gender differences were addressed to achieve an accurate 3D-representation of the spine. The approach was validated by comparing the estimated positions of the sensors on 14 healthy subjects with those provided by an optical motion capture system. A mean position error of lower than 12 mm was obtained, thus showing that the proposed method can offer an effective non-invasive tool for the assessment of spine posture.

## 1. Introduction

The spine is defined by a strong, stable and flexible structure composed of vertebrae, [1] and its shape is optimized for erect posture [2]. Incorrect posture and spinal abnormalities and deformities may modify the stability of this structure and its load distribution, which can generate back pain and injuries, thus provoking a reduction in quality of life [3] and an increased risk of mortality in people with vertebral fractures [4]. For this reason, accurate determination of spine posture is of great interest for the effective prevention, evaluation, treatment and evolution monitoring of spinal disorders. A correct spine posture determination means a correct determination of the position and orientation of vertebrae, so that a diagnosis is enabled. However, the complex and subject-specific human anatomy makes the accurate determination of the three-dimensional (3D) spine posture using non-invasive techniques difficult.

The plain radiography image remains an essential tool for the diagnosis of spinal abnormalities and deformities, and it is still used as the gold standard [5]. It involves sending ionizing radiation (X-rays) through the body to produce a two-dimensional (2D) exposed digital image [6]. The EOS^®^ imaging system offers the possibility of obtaining 3D information from two X-ray images, by placing two pairs of tubes and detectors orthogonal to each other [7]. An axial computed tomography (CT) scan uses a rotating X-ray tube to provide multiple cross-sectional images of the body, which allows a 3D reconstruction of human bodies. However, because radiation exposure increases the risk of cancer [8], the use of X-ray technologies is limited, and they must be performed in a specialized room in order to block radiation exposure to others. While magnetic resonance imaging (MRI) offers a 3D observation without radiation exposure, in addition to its limited projection volume, its accuracy is dependent on the operator’s skill and the degree of immobility of the subject. Moreover, in most CT scan and MRI systems, the subject is placed in a supine position and, consequently, their erect posture cannot be analyzed. Finally, CT ($1 M), MRI ($2 M) and EOS ($0.5 M) technologies are expensive and often not affordable for a small business [6,9].

In addition to the invasive approach offered in [10], there is a wide range of non-invasive assessment tools to visualize spine posture currently available for clinical use [11]. Even though optoelectronic systems can yield very accurate results, the use of skin-attached markers does not usually offer information on spine rotations [12]. Recent developments in microelectromechanical systems have caused a renewed interest in the use of inertial measurement units (IMUs) to record human posture in 3D [13,14,15,16,17]. IMUs are capable of estimating their own orientation within an Earth-fixed frame using sensor fusion algorithms, such as Madgwick’s algorithm [18] or the extended Kalman filter (EKF) [19]. These algorithms provide an estimate of the orientation by combining the information sourced from the triaxial accelerometer, gyroscope and magnetometer present in the IMU. Although these tools are affordable for clinical use, the cited studies only determined the shape of the surface profile, and most of them were only in 2D. Even though it has been demonstrated that spinal curvature and skin surface curvature present similarities in the sagittal plane [20,21,22], the relationship between the skin profile and the vertebral body is not trivial. This is due to the variable longitudinal displacement (in the posterior–anterior direction) between the spinal curve and the skin surface curve, induced by the spinous processes and the soft tissues [20,22], which introduces angular offsets in the sagittal plane between the external measurements and the internal vertebrae orientations, as well as scaling difficulties. Only Butnariu and Antonya have considered the displacement between both curves [13], but they limited the application of the displacement correction to a 2D curve, based on measurements from inertial sensors attached to a flexible bar, without paying attention to angular compensations or scaling issues. Moreover, to offer a 3D visualization, their scaled 3D models of vertebrae were located and oriented according to the corrected 2D curve, but they did not generate a multibody system (a set of rigid or flexible bodies connected by joints), to allow the study of the dynamic behavior of the interconnected bodies for load estimations. The correct three-dimensional location and orientation of the spinal bodies are essential factors to accurately predict vertebral loading, because it is influenced by the curvature of the spine [23].

Since spinal curvature is soft (it can be approximated by a cubic spline [24]), and the relative orientations of the vertebrae are limited by anatomical restrictions [1], with the help of a multibody model of the spine, a reduced number of orientation sensors can be used to estimate a higher number of vertebral orientations by interpolation. In this work, a procedure to obtain a subject-specific multibody model of the spine using any type of 3D orientation sensors was proposed, and, based on this multibody model, an estimation of the locations and orientations of the 17 vertebrae constituting the thoracolumbar spine was made. Inertial sensors and clusters of three markers located with an optical motion capture system were both used and validated as the 3D orientation sensors. The number and calibration of the sensors, angular offsets, scaling difficulties and gender differences were addressed to achieve an accurate 3D-representation of the spine. As was accomplished in a previous work [25], to validate the approach and to evaluate accuracy and consistency issues due to the IMU measurements (closely related to sensor calibration and magnetometer sensitivity [26,27,28]), three optical reflective markers were attached to each inertial sensor. The approach was validated by comparing the estimated position locations of the sensors on 14 healthy subjects with those directly provided by an optical motion capture system. Additionally, we have shown that the proposed method can be applied using the orientations obtained from the markers, instead of using the orientations provided by the IMUs, thus offering a new configuration of markers to estimate the 3D-representation of the spine, and we offer an evaluation of the proposed approach with a more accurate measurement system.

The remainder of the paper is organized as follows: Section 2 describes the experiments carried out, provides details on the two essential components of the proposed method (the sensors and the spine multibody model), and explains the method itself. Section 3 shows the results from applying the proposed method to the 14 volunteers, obtaining the data for the orientation of the inertial sensors either from their own readings or via optical means, and compares them with those directly provided by the optical system, thus evaluating the accuracy of the proposed method. Section 4 discusses the errors obtained and the optimal sensor configurations as functions of the number of sensors used, highlights the key aspects of the proposed method, and indicates pending developments. Finally, Section 5 summarizes the main conclusions of the work.

## 2. Materials and Methods

**Figure 1:**
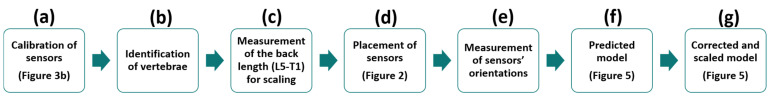
Flowchart of the full procedure.

To facilitate understanding of the proposed method that is described in this section, a flowchart is presented below (Figure 1).

### 2.1. Experimental Data Collection

**Figure 2:**
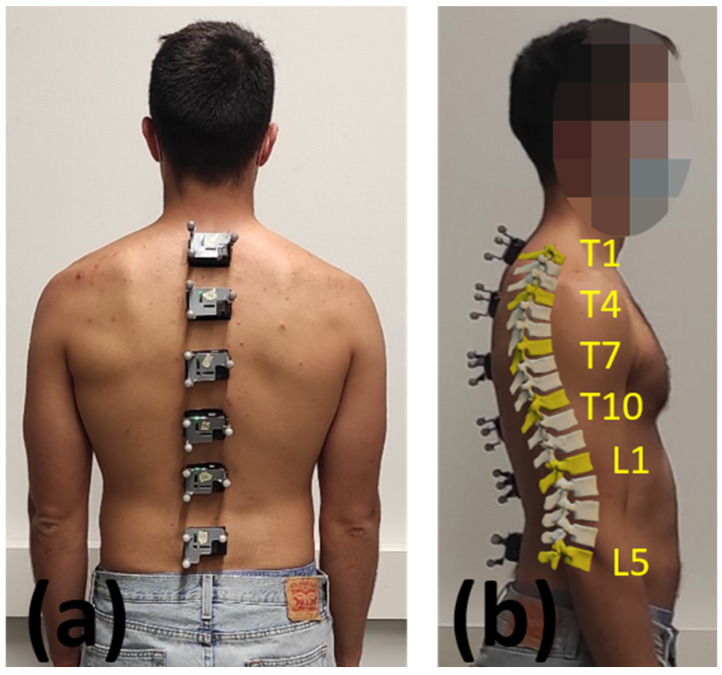
(**a**) Sensors attached to the subject’s body (back view); (**b**) Sensors attached to the subject’s body (lateral view).

**Figure 3:**
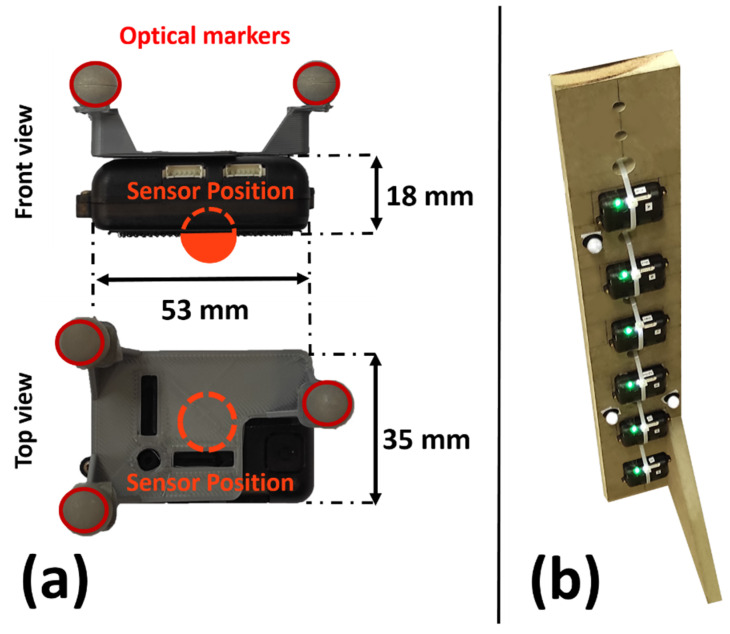
(**a**) IMU dimensions and marker attachment; (**b**) Wooden plate for calibration of the inertial sensors.

Fourteen volunteer subjects (7 males, 7 females, age 41 ± 12 years, height 172 ± 19 cm, body mass 67 ± 20 kg) were recruited for this study, all of whom had no history of spinal surgery, tumors or disorders of the trunk. All subjects gave written informed consent for their participation. Six IMUs (STT-IWS, STT Systems, San Sebastian, Spain) sampling at 100 Hz, were attached to the subject’s body with double-sided adhesive tape at vertebrae Thoracic 1 (T1), Thoracic 4 (T4), Thoracic 7 (T7), Thoracic 10 (T10), Lumbar 1 (L1) and Lumbar 5 (L5) (Figure 1d), as can be seen in Figure 2. Vertebrae were identified by a physiotherapist with the subjects in the standing position (Figure 1b). Three optical markers were fixed to each IMU (Figure 3a) in order to provide an alternative way of determining its position and orientation, and the data was computed by 18 OptiTrack FLEX 3 infrared motion capture cameras (Natural Point, Corvallis, OR, USA), also sampling at 100 Hz. The distance along the skin surface of the back from T1 to L5 (43 ± 4 cm) was measured using a soft sewing measure to scale the multibody model of the spine to the individual subject (Figure 1c). Participants were asked to stand still for a few seconds in order to record the synchronized data from both motion capture systems (optical and inertial) (Figure 1e). The orientations of the IMUs were computed by an in-house-developed Madgwick’s algorithm [18]. This algorithm used a quaternion representation, allowing accelerometer and magnetometer data to be used in an analytically derived and optimized gradient descent algorithm to compute the direction of the gyroscope measurement error as a quaternion derivative.

### 2.2. Sensor Orientation and Geomagnetic Frame of Reference

As explained in [28], the optical and inertial systems each use a different global reference frame. Although this frame should be the same for all IMUs, their inherent errors in determining the directions of gravity and magnetic North lead to discrepancies among different sensors. For this reason, the initial relative offsets of the sensors were estimated using a wooden calibration plate (Figure 3b), and subsequently corrected (Figure 1a). The applied correction was valid for a whole day of experimental measurements. The axes of the global reference frame of the optical system were defined by the calibration process as follows: *x*-axis in the posterior–anterior direction, *y*-axis in the medial–lateral direction, and *z*-axis in the vertical direction. Once calibrated, the global reference frames of the sensors shared the same vertical *z*-axis, but their *x*-axes were given by the direction of magnetic North. Therefore, to compare the data obtained by both motion capture systems, the initial *z*-axis rotations of the base body (L5), as measured by both the optical system and the inertial sensors, were canceled in the orientations of both the markers and the IMUs.

### 2.3. Spine Multibody Model

**Figure 4:**
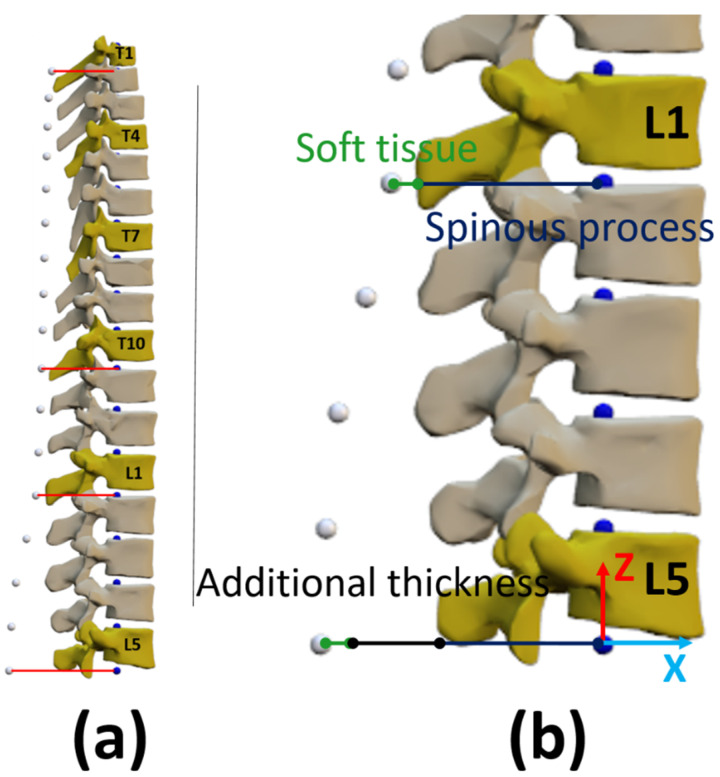
(**a**) Multibody model of the human thoracolumbar spine; (**b**) Longitudinal displacement. Blue dots: origins of local reference systems of vertebrae. White dots: estimated skin surface.

The human thoracolumbar spine was modeled as a three-dimensional multibody system formed by rigid bodies. The model consisted of 17 anatomical segments: 12 thoracic vertebrae and 5 lumbar vertebrae (Figure 4a). The origin of the base body, L5, was fixed (because IMUs do not provide positions) and segments were linked by ideal spherical joints, thus defining a model with 51 degrees of freedom (DOFs). The estimated skin surface, corresponding to the external surface of the back where the sensors were attached, was obtained by applying a displacement along the local *x*-axis (local posterior–anterior direction) of the vertebra frame of reference (Figure 4). The total posterior–anterior displacement (red lines in Figure 4a) was composed of the length of the spinous process and the thickness of the soft tissue [20,22] (Figure 4b). The spinous process length was measured in the multibody model at T1 (3.1 cm), T10 (4.6 cm), L1 (5.1 cm) and L5 (4.5 cm) (blue lines in Figure 4b). Then, the thickness of the soft tissue (green lines in Figure 4b) of 1.35 cm [22] was added to each of these vertebrae, and, because the lower lumbar region, particularly at L5, presents a thicker region of soft tissue due to the presence of both muscle and adipose tissue around the upper gluteal muscles [22], an additional thickness of 1.7 cm was added to L5 (black line in Figure 4b). Finally, the estimated skin surface was interpolated with a cubic spline. The resulting model of the spine was then adjusted and scaled to each subject as described below.

### 2.4. Predicted Vertebral Orientations

**Figure 5:**
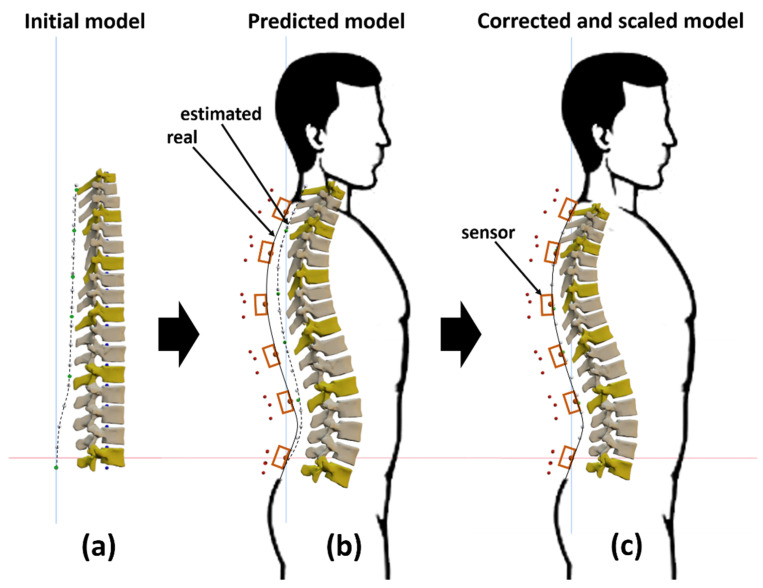
Posture determination process: (**a**) initial model; (**b**) predicted model; (**c**) corrected and scaled model.

As might be expected of any non-invasive device, the objective of this tool was to estimate the relative positions and orientations of the internal bodies constituting the spine from the external information taken on the surface of the skin. Because the length of the skin surface is affected by the spine curvature, before scaling the model with the measured skin surface length, the bodies need to be correctly oriented. Although spinal curvature and skin surface curvature present similarities [20,21,22], angular offsets in the sagittal plane are induced by the variable longitudinal displacement between both curves [20,22]. These angular offsets are proportional to the relative rotations of the vertebrae, so they are subject-specific, and must be determined for each subject individually in order to correct the measurements. To estimate the angular offset, the spine bodies were first oriented and positioned (Figure 1f) using the direct measured orientation of the sensors (Figure 5b).

Because the spinal curvature is soft (it can be approximated using a cubic spline [24]) and the relative orientations of the vertebrae are limited by anatomical restrictions [1], a reduced number of sensors can be used to estimate a higher number of vertebral orientations by interpolation. To obtain the orientations of the 17 bodies using a reduced number of sensors, the measured yaw, pitch and roll angles were interpolated with a piece-wise cubic polynomial, by means of Matlab’s function *pchip*. In this work, the interpolated angles were estimated from several sensor configurations, varying the number of the orientations measured by sensors from 4 to 6. The proposed procedure to obtain a subject-specific spine multibody model can be applied using the 3D orientations of the sensors from any system. In this work, we used both inertial sensors and clusters of three optical markers to provide the orientations of the sensors.

Because the anteversion of the pelvis induces a higher inclination of the attached lumbar L5 in the sagittal plane, an additional rotation of 10° for men or 14° for women was added. The differences between the sexes for the angle used in this work corresponds to the differences in the acetabular version reported in the literature [29,30,31].

**Figure 6:**
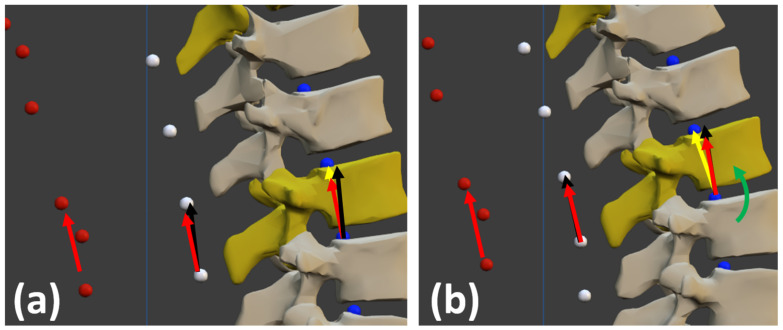
(**a**) Angular offset between the orientation measured by the sensor (red vectors) and the external segments defined by the estimated external surface of the back (black vectors); (**b**) Angular offset and corrected body orientation (yellow vector) by rotation (green arrow).

From this predicted spinal curvature, the angular offsets were defined as the angles between the orientation measured by the sensor (red vectors in Figure 6) and that of the external segments (black vectors in Figure 6). The external segment of a vertebra corresponds to the vector defined by the estimated skin surface attached to this vertebra and the estimated skin surface attached to the superior vertebra.

### 2.5. Corrected Vertebral Orientations and Scaling

The estimated angular offsets were then subtracted from the sensor measurements, and the spine bodies were newly oriented and positioned by interpolating the corrected measured orientations. Because the bodies were now correctly oriented, the scale factor could first be determined as the ratio between the measured distance along the real back skin surface on the subject (from T1 to L5) and the calculated distance along the estimated one (Figure 5b), and then be applied to the dimensions of the bodies: vertebral body height, spinous process length and soft tissue thickness (Figure 1g).

From here on, the proposed method used to build a personalized multibody model of the subject’s spine based on the orientations of several inertial sensors placed on various identified vertebrae, and to estimate the spinal posture from this personalized multibody model, is called TPM. Moreover, where the orientations of the inertial sensors were provided by the inertial sensors themselves, the method is called TPM-IMU, and where the orientations of the inertial sensors were obtained using the cluster of three markers attached to each sensor and the optical motion capture system, the method is called TPM-OPT.

## 3. Results

**Figure 7:**
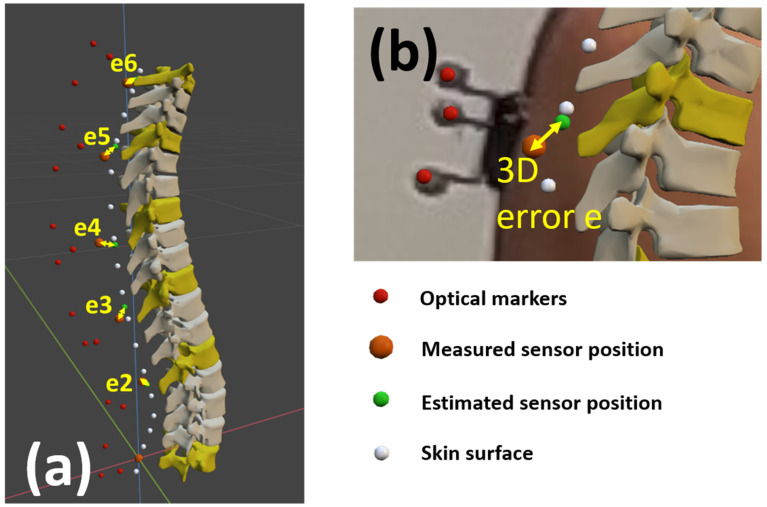
(**a**) Spine multibody model; (**b**) Detail and meaning of the colored dots.

To validate the proposed method and the correct location of the bodies, the estimated positions of the sensors (green dots in Figure 7) obtained by applying TPM (either TPM-IMU or TPM-OPT), were compared with those directly measured by the optical system (orange dots in Figure 3a and Figure 7); i.e., by using the coordinates of the markers attached to each sensor (red dots in Figure 7) provided by the optical motion capture system and calculating from them the coordinates of the desired location point for the sensor. The estimated positions of the sensors (green dots in Figure 7) corresponded to the estimated extreme points of the corresponding spinous processes, as the sensors were located at vertebrae identified by a physiotherapist based on the palpation of the spinous processes.

The origin of the two systems (optical and inertial) was set at the position of the first IMU at L5, and the distances between the measured and the estimated positions of sensors #2 to #6 (e2 to e6 in Figure 7) offered five check-points to evaluate the curvature precision and to validate the approach.

The position errors corresponding to IMUs #2, #3, #4, #5 and #6, were evaluated for the 14 subjects using both TPM-IMU and TPM-OPT, and different sensor configurations (Table 1). 

Using TPM-OPT, the mean errors were 11.6 mm for 6 sensors, between 11.9 mm and 14.1 mm for 5 sensors, and between 13.6 mm and 26.3 mm for 4 sensors, respectively. The use of TPM-IMU showed similar values: the mean errors were 12 mm for 6 sensors, between 12.5 mm and 13.7 mm for 5 sensors, and between 13.7 mm and 24.6 mm for 4 sensors. The mean error position at T1 (e6), the last body of the open kinematic chain, was 8.3 mm for 6 sensors with TPM-OPT, and, with fewer sensors, e6 was found to be lower than 10.3 mm when using the optimal configuration. As the mean measured distance along the skin of the back for all subjects was 43 cm, the error at T1 was lower than 2%.

**Figure 8:**
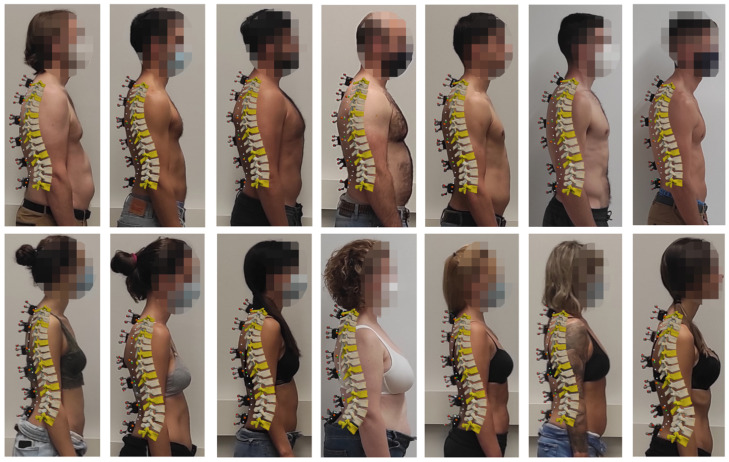
Lateral view of the fourteen 3D models obtained with TPM-OPT and six sensors.

The fourteen 3D models obtained using TPM-OPT and six sensors are represented in Figure 8 (lateral view). Marker positions (red dots) were used to fit the 3D model to the subject’s picture. The seven images in the first row are from men, and the seven images in the second row are from women. It can be observed that the curves defined by the estimated sensor positions (green dots) and skin surface (white dots) match well with the curves defined by the measured sensor positions (orange dots) and the actual subject’s back, regardless of the size and shape of the spine.

**Figure 9:**
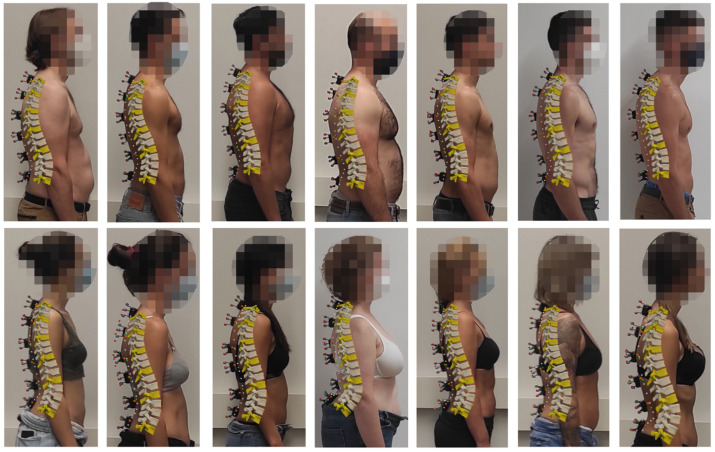
Lateral view of the fourteen 3D models obtained with TPM-IMU and six sensors.

The fourteen 3D models obtained using TPM-IMU and six sensors are represented in Figure 9 (lateral view). The same observations can be made again: the curves defined by the estimated sensor positions (green dots) and skin surface (white dots) match well with the curves defined by the measured sensor positions (orange dots) and the actual subject’s back, regardless of the size and shape of the spine. Moreover, comparing Figure 8 and Figure 9, it can be seen that differences between TPM-OPT and TPM-IMU are almost undetectable by visual inspection.

## 4. Discussion

This work proposed an approach to determine the 3D human spine posture through a multibody model using the orientations of a set of sensors attached to the back of the subject, obtained either by the readings of inertial sensors (TPM-IMU) or by an optical motion capture system where the sensors were simply clusters of markers (TPM-OPT). 

The calculation of the differences between the direct optical measurement of the positions of the sensors (from the three markers attached to each of them) and the estimated position of the sensors, using either TPM-OPT or TPM-IMU for positions #2 to #6, served to validate the approach. The errors obtained with TPM-OPT showed a mean value lower than 12 mm using either six or five sensors. The mean errors obtained with TPM-IMU were 12 mm using six sensors and 12.5 mm using five sensors. Differences between the two procedures were almost undetectable by visual inspection (Figure 8 and Figure 9). For both methods, the optimal configuration for 5 sensors was L5-L1-T10-T7-T1, and for 4 sensors it was L5-L1-T10-T1. As expected, using the optical system to obtain the orientations of the sensors (TPM-OPT) offered smaller mean positional errors than using the inertial sensors for the same purpose (TPM-IMU) and, by increasing the number of sensors, the accuracy increased. 

The mean error e6 at the top of the thoracolumbar spine (T1) was lower than 9 mm using 6 sensors (TPM-OPT and TPM-IMU), which corresponds to 2% of the subjects’ mean skin surface length. At first glance, it could be surprising that errors e2 (L1) and e3 (T10) were higher than e6 (T1). The reason is that, while these points are closer to the origin of the model (L5), the lumbar region is affected by a thicker region of soft tissue due to the presence of both muscle and adipose tissue around the upper gluteal muscles [22], and the anteversion of the pelvis induces a higher inclination at the first lumbar vertebrae, thus making the accurate determination of the position of this region more difficult. 

The complex mathematical model proposed in [16] to reproduce the shape of the spine showed position errors of 5 mm, but it only defined the shape of the skin surface of the back in 2D instead of the full 3D representation of the spine proposed here, and the positions of the sensors were precisely known. It must be highlighted that validation of the results in this work was doubly affected by any errors in the identification of vertebrae. First, because the orientation of the corresponding body will be wrong, and second, even if the curvature is not significantly affected, incorrect vertebral identification will proportionally increase the distance error. 

Owing to the additional rotation imposed at L5, the curves defined by the estimated skin surface matched well with the curves defined by the optical markers for both sexes. Correct location and orientation of the spine bodies are of great interest, because spine curvature influences vertebral loading and risk factor patterns for neutral standing and other activities [23], which means that the approach offered in this work allows us to accurately predict vertebral loading. The procedure to estimate spine posture was validated in 3D with healthy subjects. Only lateral views are shown because the subjects did not show specific deformities in the back view. However, it must be noted that spinal abnormalities and deformities could alter the correspondence between the external surface of the back and the underlying bones.

Future work includes: (i) to validate the proposed method against X-ray imaging, the gold standard; (ii) to test a broader group of people, considering subjects with lateral deformities, such as scoliosis, and subjects of different ages, body fat percentages, etc., since the relationship between the external and internal curves requires corrections which depend on the subject’s personal characteristics; and (iii) to study the accuracy provided by the proposed method when measuring the spine configuration along the motion, as the skin artifact can affect the location of sensors with respect to their associated vertebrae [32].

## 5. Conclusions

The conclusion is twofold. First, it has been shown that wearable inertial sensors offer a non-invasive tool for the assessment of spine posture which can be valuable for clinical use. Second, a procedure has been proposed to obtain a subject-specific spine multibody model for computational simulations using any type of 3D orientation sensors. In this work, both inertial and optical sensors have been tested.

## Figures and Tables

**Table 1:** Position error, in mm, between estimated (TPM-OPT or TPM-IMU) and measured (direct measurement with optical system) sensor positions.

		Position Error (mm)											
		e2		e3		e4		e5		e6		Mean	
# Sensors	Positions	OPT	IMU	OPT	IMU	OPT	IMU	OPT	IMU	OPT	IMU	OPT	IMU
**6**	**L5-1-T10-7-4-1**	13.4	14.3	14.3	14.0	12.0	12.9	12.0	10.3	8.3	8.7	11.6	12.0
**5**	**L5-1-T10-7-1**	13.5	14.2	14.3	13.9	12.0	12.8	12.0	10.3	10.3	11.0	11.9	12.5
**5**	**L5-L1-T10-4-1**	13.4	14.3	14.2	14.1	11.9	12.5	11.9	13.3	13.0	11.6	13.5	13.2
**5**	**L5-1-T7-4-1**	13.3	14.2	17.1	15.7	16.5	16.7	16.5	12.4	10.4	9.5	14.1	13.7
**4**	**L5-1-T7-1**	13.0	14.3	13.9	15.6	14.0	18.1	14.0	17.4	16.9	22.3	14.2	17.5
**4**	**L5-1-T10-1**	13.3	14.3	17.3	15.9	17.0	17.4	17.0	10.6	10.2	10.5	13.6	13.7
**4**	**L5-1-T4-1**	13.3	14.1	22.3	20.0	29.3	27.5	29.3	31.7	32.2	29.6	26.3	24.6

## Data Availability

The data presented in this study are available on request from the corresponding author. The data are not publicly available due to privacy restrictions.
